# Static Posturography: A New Perspective in the Assessment of Lameness in a Canine Model

**DOI:** 10.1371/journal.pone.0170692

**Published:** 2017-01-23

**Authors:** Maria E. Manera, José M. Carrillo, Miguel Batista, Monica Rubio, Joaquin Sopena, Angelo Santana, José M. Vilar

**Affiliations:** 1 Departamento de Patología Animal, Instituto Universitario de Investigaciones Biomédicas y Sanitarias, Universidad de las Palmas de Gran Canaria, Arucas, Las Palmas, Spain; 2 Departamento Medicina y Cirugía Animal, Cátedra García Cugat, Universidad CEU Cardenal Herrera, Valencia, Spain; 3 Departamento de Matemáticas, Universidad de las Palmas de Gran Canaria, Las Palmas, Spain; Northwestern University, UNITED STATES

## Abstract

The aim of this study was to assess the static posturography in dogs as a useful tool for diagnosis of lameness by means of the use of a pressure platform. For this purpose, a series of different parameters (pressure distribution, area of support, mean pressure, maximum pressure and statokinesiograms) were obtained from five lame dogs with unilateral elbow osteoarthritis treated with plasma rich in growth factors. Data were obtained before and 3 months after treatment, and results were compared with a control group of sound dogs of similar conformation. Significant differences were found in the above mentioned parameters between sound and lame limbs. Improvement after 3 months of treatment was also detected, demonstrating that this multi-parametric technique is an effective and reliable method for the assessment of lameness in dogs.

## Introduction

Peak vertical force (PVF) and vertical impulse (VI) are two of the most common kinetic parameters used for lameness detection in dogs, horses and other domestic animal species [1]; these parameters are usually obtained using force [2] or pressure platforms [3–5].

Pressure platforms, with their multiple sensors, have the potential to provide more information than force platforms; however, references describing the use of pressure platforms remain scarce, and the majority of these studies are descriptive. Previous studies describe distribution of force in the pads during the support phase in healthy dogs [6,7] or in dogs with pathologies such as cranial cruciate ligament rupture [8] or hip fractures [9]; the dogs walk or trot across a simple or multiple pressure walkway that provides standard parameters as PVF and VI, usually measured by force platforms. The advantage of this method is that consecutive steps can be recorded; however, research has still not been published on static analysis in lame dogs with postural changes, such as spatial modifications in body center of pressure (COP) and the derived consequences of changes in paw area, and mean or maximum pressure values, among other parameters.

The correct balance and its continuous preservation is a combined process connected with the central nervous system, sight, and muscular system [10]. In quiet stance position, the control of body posture is assumed as a constant action of stabilization of a multilink inverted pendulum [11,12], which corresponds with the attempt of to keep the center of mass (COM) symmetrically to the support base [13]. As posture is being constantly perturbed by internal and external mechanisms, the balance recovery is performed by constant compensatory movements, known as postural sway. In this way, static posturography becomes an objective evaluation method of the balance system, and it is widely used in human medicine [14], rehabilitation [15] or sport fields [16].

Many clinical practitioners assume that the COP position coincides with the projection of the COM on the support surface, although these two parameters are based on different concepts [17]. This sway is registered in both the latero-lateral (X) and craniocaudal (Y) axes of the body. Based on these principles, we could obtain two different graphical recordings: the statokinesiogram, which depicts the movement of COP in an X-Y coordinate system, and the stabilogram, representing the location of the COP as a time function, where movements in the X and Y axes are considered separately.

When lameness is present, the associated pain causes loss of balance in the static position and is provoked by the patient transferring weight from the painful limb to the healthy (or less lame) contralateral in an effort to alleviate the pain [18]. In other words, pain can also cause postural (COP) modifications. Clinically, impaired COP balance returns to a more normal value in some cases when an effective treatment is applied; this suggests that changes in COP balance could also be a predictive tool for gait recovery [19], thus meriting evaluation [20]. In addition, theoretically, changes in COP balance in lame dogs should determine changes in paw area, as the pads are elastic structures that spread when ground contact pressure grows [21,22]. The patterns of pressure distribution in the paws might also be assessed to generate useful, objective, and complementary data as location of maximal pressure point and/or limb COP location within the paw to evaluate locomotor system status.

The term medial coronoid disease (MCD) encompasses all pathologic changes of articular cartilage and subchondral bone involving the medial coronoid process of the elbow joint [23,24]. MCD is the most common cause of thoracic limb lameness in large and giant breed dogs [25], and MCD lesions have traditionally been evaluated radiographically [26–28]. A presumptive diagnosis of MCD is frequently based on detection of the resultant secondary osteoarthritis (OA), rather than on detection of the primary lesion [26,29].

Different strategies have been proposed for the treatment of OA, and among them, Platelet Rich Plasma (PRP)-based products such as plasma rich in growth factor (PRGF) therapy, has been widely used as a single or co-adjuvant therapy in the treatment of OA in dogs [30]. Based on this, we hypothesized that assessment of COP variations, together with other secondary static parameters, could serve as an objective and quantifiable tool to detect lameness and its variations.

The aim of this study was to test static posturography as a potentially reliable, objective method to evaluate lameness in five lame dogs affected by OA in elbow joints. The verification of this objective builds a foundation for the feasibility of using static posturography in the clinical assessment of postural stability in lame dogs.

## Materials and Methods

### Animals

A total of 10 client-owned, adult dogs of similar conformation were used in this study. The body weight of enrolled dogs ranged from 30 to 41.8 kg, and ages were 3 to 9 years.

The control (sound) group was formed by five dogs (two Labradors and three Rottweilers). As they were sound dogs, a certain asymmetry should be assumed; for that reason, for comparison purposes, limbs with lesser and higher values were specifically considered. The study group contained five lame dogs (three Labradors and two Rottweilers). The lameness was unilateral and clinically classified as “severe” although with presence of weight bearing in all dogs and attributable to OA secondary to MCD of the elbow joints. Dogs did not receive medication of any kind over the 4 weeks before the analysis.

To confirm or rule out OA, three standard radiographic views of both elbow joints (a lateral extension, lateral flexion, and a 15° oblique craniomedial caudolateral) [24] were taken under sedation with dexmedetomidine 10–20 μg/kg (Dexdomitor, zoetis, Spain)from dogs belonging to both study and control groups. Additional standard radiographs of knee and hip joints were taken in order to ensure that elbow OA was the unique reason for the observed clinical signs. A complete clinical evaluation (physical examination, including vital signs and neurologic and orthopedic exams) assured that general health was otherwise normal.

The procedure was revised and authorized by the Ethical Committee of Animal Welfare (CEBA) of the University CEU Cardenal Herrera of Valencia. The owners of each animal gave permission and signed a written consent form.

### Obtention-inoculation of PRP

PRP was obtained using similar procedure to the PRGF^®^ (BTI, Vitoria, Spain), but with different materials. This product produces a moderated amount of platelets (double respecting to peripheral blood) and less than 0.3 leukocytes/ μL. The procedure is as follows: whole blood (10 mL) was aseptically extracted from the cephalic vein and collected in two 4.5-mL centrifuge tubes (BD Vacutainer^®^, Plymouth, UK), each containing 0.5 mL citrate solution, then centrifuged for 8 minutes at 460 × *g*. Only the inferior third of the obtained plasma (adjacent to the buffy coat) was used to be activated with 5% of its volume with 10% calcium chloride. The resultant ~2 mL solution was injected aseptically into the elbow joint through the conventional arthrocentesis site previous sedation with dexmedetomidine iv. The appearance of joint fluid confirmed proper needle placement. A total of four doses were administered on D0, D7, D14, and D21. After every inoculation, exercise was restricted to a walk of maximum of 30 minutes at leash during the following 2 days.

### Static posturography

For the recording of data, a pressure platform (EPS/R1, Loran Engineering, Bologne, Italy) was used. The device contains a total of 2096 pressure sensors of 1 cm^2^ distributed in an area of 48 × 48 cm. The range of pressure was 30–400 kPa, and acquisition frequency 100 Hz. Animals were placed in quiet stance with their thoracic limbs over the pressure platform, perpendicular to the ground, and each dog’s owner remained in front of the animal to attract the dog’s attention at a close distance. Three recordings of 10 seconds were obtained from each animal. Only recordings in which the animal was completely immobile in symmetric position were considered valid.

Data acquisition was performed using Biomech software (Loran Engineering). The parameters for static posturography were as follows:

- Distribution of pressure between both limbs measured in kilopascal (Kpa) and expressed in percentage.
- Changes in paw area measured in cm^2^ and expressed in percentage.
- Mean and maximum pressure of each thoracic limb measured in Kpa.
- Graphic distribution of pressure ranges in paws of lame and sound limbs shown in a 2-dimensional and 3-dimensional (3-D) color scale from blue (low pressure), to red (high pressure). In order to obtain a correct contrast among the colors in the different pressure ranges, calibration was set manually to 212 Kpa to avoid saturation of the sensors.
- Statokinesiogram represents the amplitude of the spatial migration of the COP in a 2-D space, estimated by computing ellipse area (measured in mm^2^), which contains 90% of the data points of the COP trajectory.
- Stabilogram recorded independent X and Y oscillations (measured in mm) as a function of time.

### Statistical analysis

Parameters were estimated by using the free R statistical software (https://www.r-project.org/). Analysis of variance with repeated measures and a Tukey test were used to determine significant differences. Normality and homoscedasticity of residuals was confirmed using Shapiro and Levene tests, respectively.

## Results

The animals had a mean body weight of 36.88 ± 4.25 kg and a mean age of 5.8 ± 2.04 years.

The mean values± SD of all obtained parameters are summarized in Table 1

**Table 1:** Posturographic Parameters in Dogs, Expressed as Mean ± SD, and 95% Confidence Intervals.

Day	LL	SL	% Difference
**Pressure Distribution**			
**0**			
	39.72 ± 2.50%	60.28 ± 2.50%	20.56 ± 5.00%
	(38.34, 41.10)	(58.90, 61.66)	(17.79, 23.33)
**90**			
	45.75 ± 2.63%	54.25 ± 2.63%	8.51 ± 5.25%
	(44.29, 47.20)	(52.80, 55.71)	(5.60, 11.42)
**Controls**			
	48.19 ± 1.24%	51.81 ± 1.24%	3.63 ± 2.48%
	(47.50, 48.87)	(51.13, 52.50)	(2.25, 5.00)
**Paw Area (cm**^**2**^**)**			
**0**			
	38.20 ± 2.68	49.93 ± 2.63	26.70 ± 5.63%
	(36.72, 39.68)	(48.48, 51.39)	(23.58, 29.82)
**90**			
	45.93 ± 2.28	49.53 ± 1.73	7.60 ± 3.27%
	(44.67, 47.20)	(48.58, 50.49)	(5.79, 9.41)
**Controls**			
	42.93 ± 2.74	44.20 ± 2.65	2.93 ± 1.88%
	(41.42, 44.45)	(42.73, 45.67)	(1.89, 3.97)
**Mean Pressure**			
**0**			
	89.61 ± 13.79	112.11 ± 13.03	22.78 ± 5.82%
	(81.97, 97.25)	(104.89, 119.32)	(19.56, 26.00)
**90**			
	91.93 ± 7.62	101.22 ± 7.43	9.69 ± 4.16%
	(87.71, 96.15)	(97.10, 105.34)	(7.38, 11.99)
**Controls**			
	93.39 ± 9.61	96.42 ± 9.02	3.27 ± 1.81%
	(88.07, 98.71)	(91.43, 101.41)	(2.27, 4.28)
**Maximum Pressure**			
**0**			
	242.44 ± 26.84	278.76 ± 20.25	36.86 ± 20.62%
	(227.57, 257.31)	(267.55, 289.97)	(25.44, 48.28)
**90**			
	296.23 ± 14.88	324.41 ± 10.86	29.25 ± 15.47%
	(287.99, 304.47)	(318.40, 330.43)	(20.69, 37.82)
**Controls**			
	298.18 ± 34.36	359.89 ± 25.65	66.06 ± 34.13%
	(279.15, 317.21)	(345.69, 374.10)	(47.16, 84.96)
**Statokinesiogram (mm**^**2**^**)**			
**0**			
	44.71 ± 26.82		
	(29.86, 59.57)		
**90**			
	13.91 ± 4.12		
	(11.63, 16.20)		
**Controls**			
	2.17 ± 1.10		
	(1.56, 2.79)		

Day 0: Before treatment in study group; Day 90: After first application of treatment in study group; SL: Sound limb in study group or limb with higher value in control group; LL: Lame limb in study group or limb with lesser value in control group.

### Pressure distribution between both limbs

Differences between lame and sound limbs in the study group diminished significantly from before treatment (day 0) to day 90 post treatment (p < 0.001). Compared with the control group, differences in pressure distribution at day 90 were also significant (p = 0.0044). Data were normal (p = 0.24) and homoscedastic (p = 0.64)

### Changes of paw area

Differences in area between lame and sound limbs in the study group diminished significantly from day 0 to day 90 (p < 0.001). Compared with the control group, differences in paw area at day 90 were not significant (p = 0.072). Data were normal (p = 0.10) and homoscedastic (p = 0.68).

### Mean pressure

Differences in mean pressure between lame and sound limbs in the study group diminished significantly from day 0 to day 90 (p < 0.001). Compared with the control group, differences at day 90 were also significant (p < 0.001). Data were normal (p = 0.76) and homoscedastic (p = 0.72)

### Maximum pressure

Maximum pressure in sound limbs in the study group increased significantly from day 0 to day 90 (p < 0.001). Compared with the control group, differences at day 90 were also significant (p < 0.001). Data were normal (p = 0.20) and homoscedastic (p = 0.10). In lame limbs, maximum pressure also increased significantly from day 0 to day 90 (p < 0.001). However, compared with the control group, differences at day 90 were not significant (p = 0.99). Data were normal (p = 0.63) and homoscedastic (p = 0.42).

### Statokinesiogram

**Fig 1:**
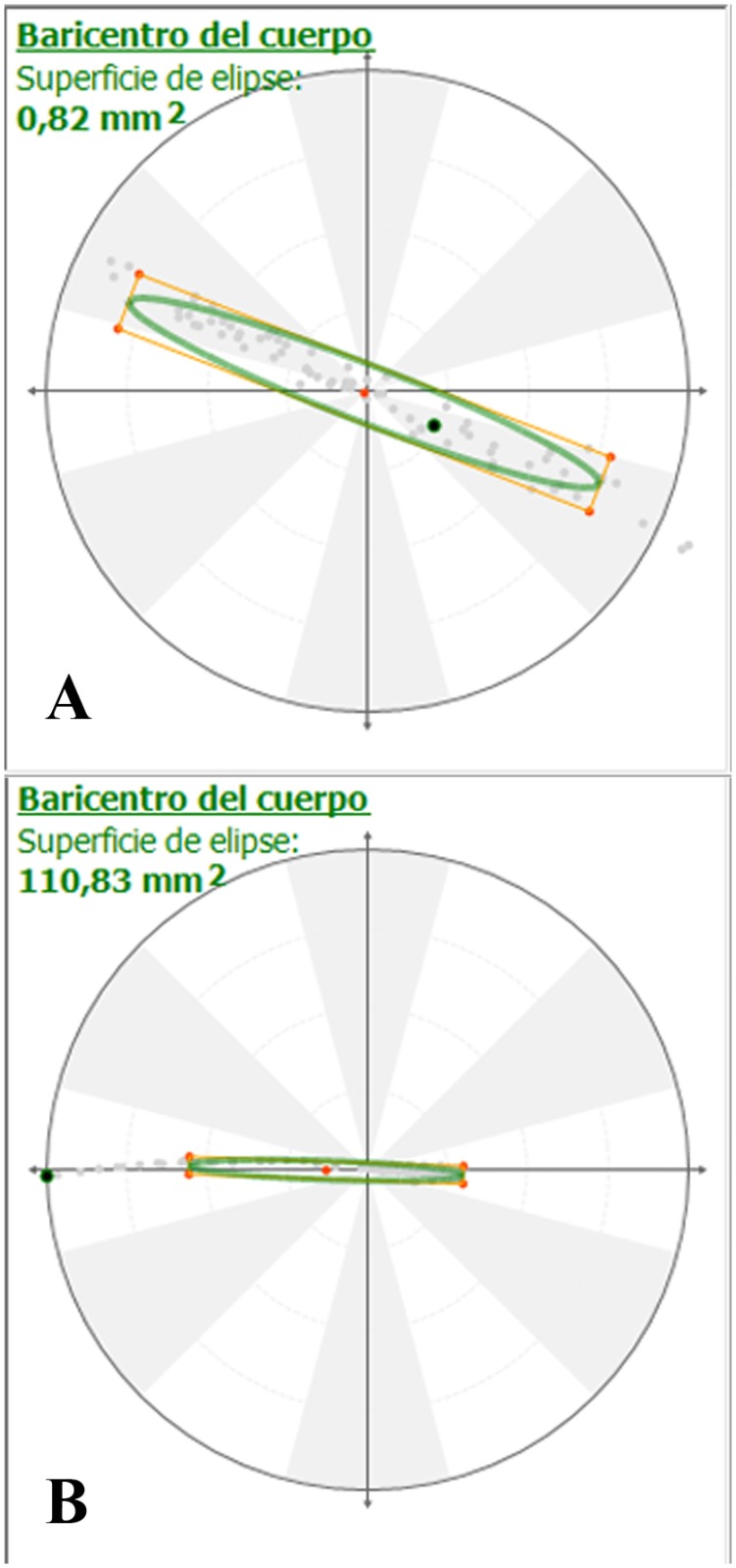
Statokinesiograms of Sound (A) and Lame (B) Dogs, Showing the Ellipse that Contains 90% of the Body COP Migration. In addition to show a greater area, in this case, a left deviation of the ellipse is evident in lame dog.

Oscillations of the COP were greater in lame dogs during the 10 seconds of recording, as observed in graphics, which sway measure area is greater in lame dogs, and assymetric (Fig 1). The sway area in the study group diminished significantly from day 0 to day 90 (p < 0.001). Compared with the control group, differences at day 90 were also significant (p = 0.005). Data were not normal (p = 0.005) but were homoscedastic (p = 0.14). For that reason, the results are reliable due to the robustness of analysis of variance when data are homoscedastic.

### Stabilogram

A symmetric latero-lateral oscillation could be seen in sound dogs, while craniocaudal oscillation was almost insignificant. In contrast, lame dogs evidenced greater or/and asymmetric oscillations.

**Fig 2:**
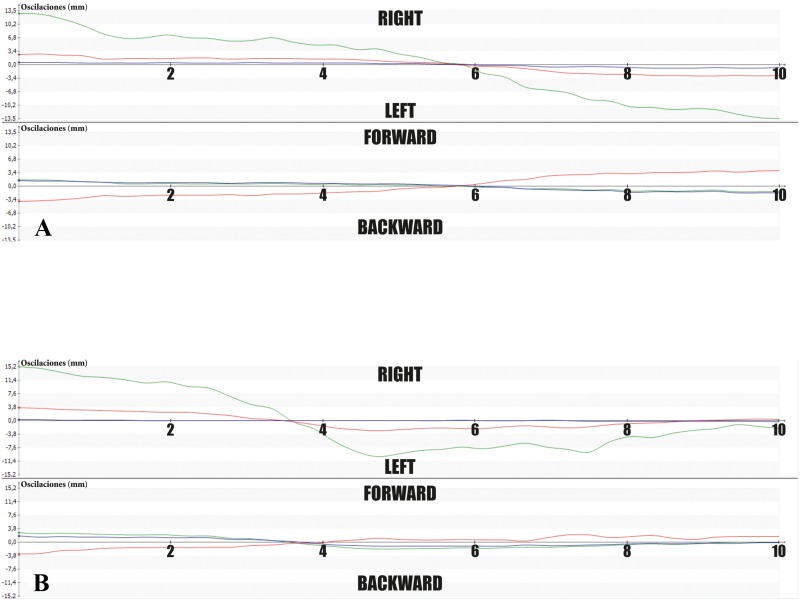
Stabilograms of Sound (A) and Lame (B) Dogs, Showing Oscillation of Body COP (Green Lines). Graphic shows that this lame dog had a greater oscillation to the right side.

Graphical representation of COP sway in X and Y axes showed asymmetry in the study group in all cases during the 10-second recording period (Fig 2).

### Graphic pressure distribution in the paws

**Fig 3:**
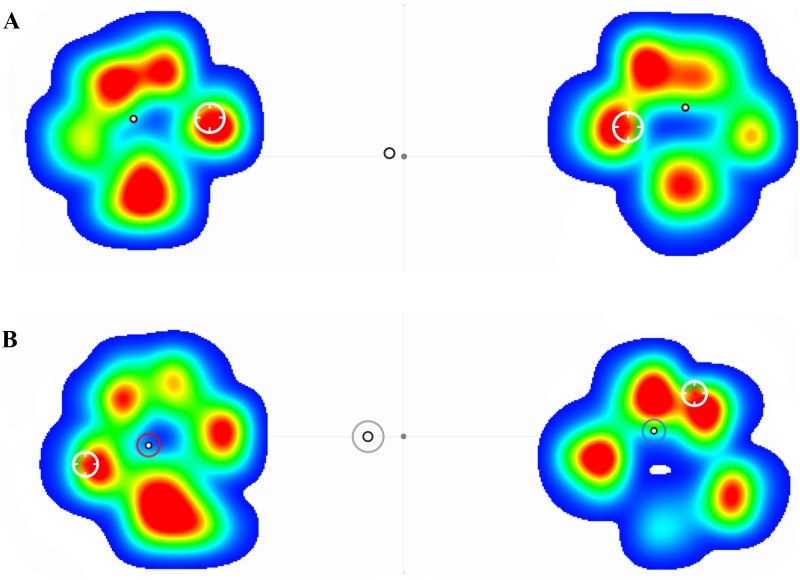
2-D Color Scale Graphics of Sound (A) and Lame (B) Dogs. In (A) symmetry is found in all measured parameters as pressure distribution, limb COP (black circle) and maximal pressure point (white circle). In (B), the pressure distribution pattern is manifestly different between lame (right) and sound limb (left), and assymetry in the other parameters is evident: limb COP (black and red in sound limb or blue circle in lame limb) maximal pressure point (white circle), body COP (black and grey circle).

2-D graphics showed as, when limbs were compared in sound dogs, a similar color (pressure) distribution pattern was evident: In addition, limb COP was symmetrically located within the paw, and the maximal pressure point was located over the 2nd digital pad. In lame group, the pressure distribution pattern when limbs were compared was markedly different, with maximal pressure point deviated laterally in the sound limb and cranially in the lame limb. Limb COP in the sound limb of lame dogs was located in the center, but in the lame limb has migrated craniomedially. The maximal pressure point was found in the sound limb laterally over the 5th digital pad, while in the lame limb, was located cranially, over the 4th digital pad. Body COP deviation towards the sound limb could also be seen (Fig 3).

**Fig 4:**
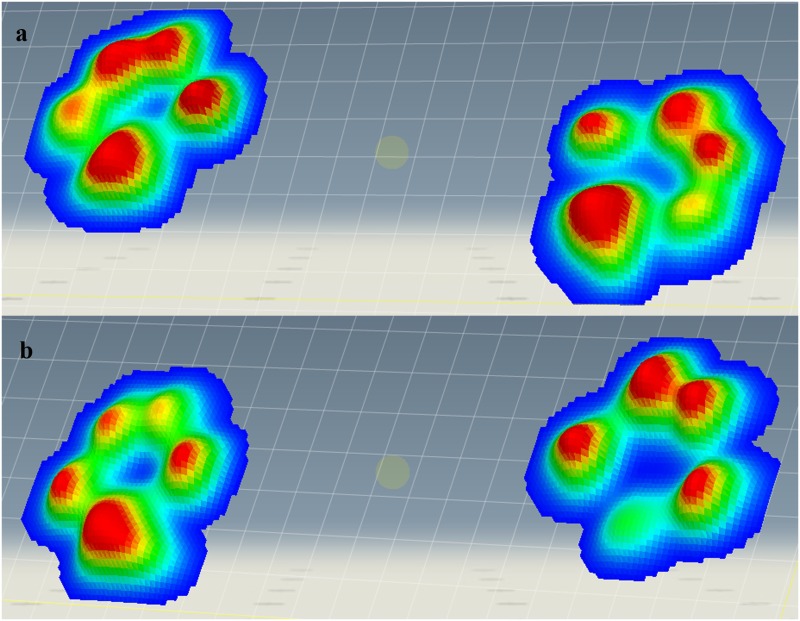
3-D Color Scale Graphic of a Sound (A) and Lame (B) Dog. In this case, lame dog shows a lateral deviation of the pressure in the sound limb (left) and a craniomedial deviation of pressure in the lame limb (right).

In 3-D color scale graphics, the pressure patterns in sound limbs were similar, while in lame limbs they are asymmetric (Fig 4).

## Discussion

With static posturography, we were able to determine differences in a set of parameters between lame and sound dogs. Additionally, these differences diminished following treatment, indicating a positive response and thus, effective therapy, as previously published in humans [31].

While the redaction of this paper was conducted, we were unable to find specific studies regarding the use of static posturography and pressure measurements of dog paws other than PVF and VI using a pressure platform. Moreover, this methodology is widely used in humans in different fields [14–16] with excellent results; this should encourage the increase of pressure platform technology use in dogs.

Statokinesiograms and stabilograms require the necessary time for recording the spatial variations of the body COP; in humans, it is advisable to calculate parameters as the average of those obtained in three successive recordings of 10–60-second duration between studies [32,33]. In agreement with these recommendations, we used three successive recordings of 10 seconds on each animal. This was enough to detect asymmetries in the dislocation of the COP.

Regarding the sampling frequency, recent observations in humans [34] suggest to consider an effective sampling rate of about 50 Hz. Considering that COP sway is a pendular movement, the pendulus length is shorter in dogs than in humans; thus, the frequency of oscillation should be, theoretically, higher. For that reason, in order to gain accuracy, we decided to increase the sampling rate to 100 Hz.

In this study, the method suitability was assessed by using dogs with forelimb lameness because the evaluation of hind limb lameness has been demonstrated harder to perform [35]. Some authors try to explain this fact arguing that the proximal joints of the hind limb are more able to decrease the supported load during the stance phase when comparing with the forelimb, as occurs in horses [36]. In addition, the lameness should be less noticeable as a lower proportion of body weight is supported by the hind limb [37].

Static posturography in dogs not only can contribute to the scientific knowledge, but also can help clinicians to graphically show lameness status to pet owners; however, some limitations were found during the design and development of this study. First, to obtain substantial differences in the parameters derived from COP migration, the use of large breed dogs was necessary. Second, it was absolutely necessary to enroll calm and obedient dogs to stand in quiet stance for the minimum time required to obtain reliable statokinesiograms and stabilograms. In addition, each trial required an adaptation phase, and during this phase, some dogs showed fatigue or lack of attention; for that reason, acquisition time was established at 10 seconds. More than this time was almost impossible to achieve in most cases.

Although 6 months is considered the minimum standard for testing the evolution of a medical or surgical treatment, we tested the animals after 3 months; we did this because after 6 months, and based on some clinical evidences, the effect of the treatment could be diminished, possibly making variation in posturographic parameters less evident or undetectable.

This study used a relative low number of animals. Although the study used dogs of similar conformation, the low sample size could result in a potential lack of statistical power. Nevertheless, we believe that this study clearly demonstrates that static posturography is a powerful tool to assess lameness in dogs and advance the objective quantification of pressure distribution anomalies in limbs due to COP sway variations. For example, in the future, the maps of pressure redistribution could help to identify and even characterize specific pathologies like those detectable using other kinematic [38] or kinetic studies [39].

Great disparity of criteria has been published about the regimen of PRP administration for OA, oscillating from one to a series of six injections [40–43]. Based on these, we decided a series of four injections with a 1-week interval between them.

## Conclusion

Based on our results, static posturography was possible to perform in dogs and provided fairly accurate measurements of various parameters not assessed before in the evaluation of forelimb lameness. These parameters, in addition to complement the classic kinetic PVF and VI, may furnish to veterinary practitioners a useful tool to assess asymmetries simultaneously due to COP sway in lame dogs.

## Supporting Information

S1 File ARRIVE guidelines. (PDF)
